# Supplementary material for: Preventing and treating PTSD-like memory by trauma contextualization
Source: Nat Commun. 2020 Aug 24;11:4220. doi: 10.1038/s41467-020-18002-w (PMC7445258; doi:10.1038/s41467-020-18002-w)
Supplement: Supplementary file 1 — Supplementary Information [file 41467_2020_18002_MOESM1_ESM.pdf]

Preventing and treating PTSD-like memory by trauma contextualization

Al Abed *et al.*

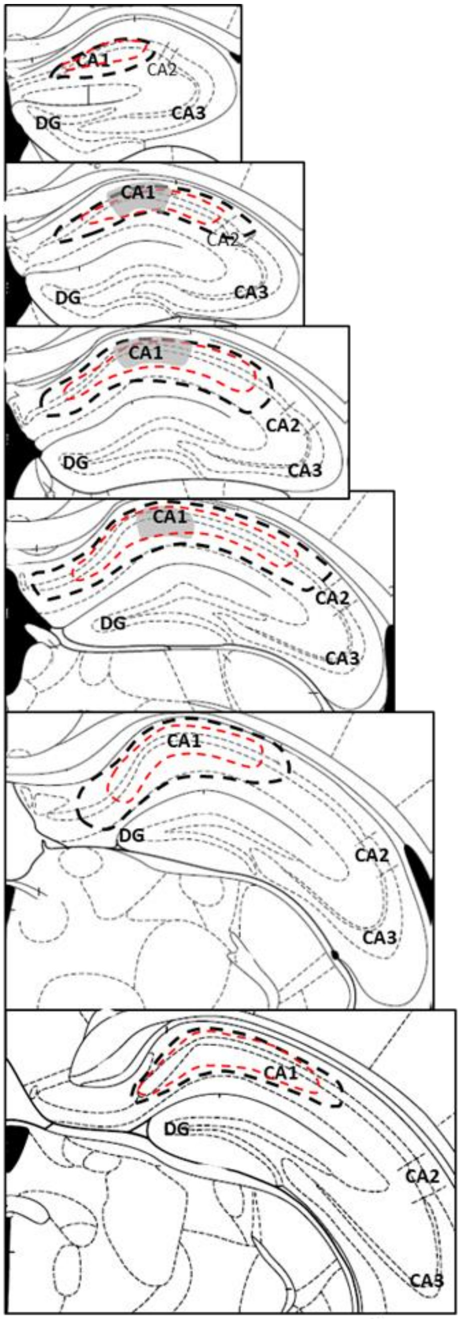

Supplementary Figure 1| Histology: Maximum and minimum areas of virus diffusion (black and red dashed lines, respectively) from the anterior dorsal CA1 (Bregma −1.34 mm) to the posterior dorsal CA1 (Bregma −2.7). The grey area represents the correct zone for optic fiber implantation. Infection or

implantation outside these marks was considered incorrect and mice were excluded from the study (From Franklin & Paxinos, 2001).

Reference:

-Franklin, K., Paxinos, G. Mouse brain in stereotaxic coordinates (Academic P. ed, Academic Press. San Diego, 2001).
