# Supplementary material for: Preventing and treating PTSD-like memory by trauma contextualization
Source: Nat Commun. 2020 Aug 24;11:4220. doi: 10.1038/s41467-020-18002-w (PMC7445258; doi:10.1038/s41467-020-18002-w)
Supplement: Supplementary file 3 — Reporting Summary [file 41467_2020_18002_MOESM3_ESM.pdf]

nature research

Corresponding author(s): DESMEDT Aline

Last updated by author(s): Jul 12, 2020

Reporting Summary

Nature Research wishes to improve the reproducibility of the work that we publish. This form provides structure for consistency and transparency in reporting. For further information on Nature Research policies, seeAuthors & Referees and theEditorial Policy Checklist .

Statistics

For all statistical analyses, confirm that the following items are present in the figure legend, table legend, main text, or Methods section.

n/a	Confirmed	
☐	☒	The exact sample size (*n*) for each experimental group/condition, given as a discrete number and unit of measurement
☐	☒	A statement on whether measurements were taken from distinct samples or whether the same sample was measured repeatedly
☐	☒	The statistical test(s) used AND whether they are one- or two-sided<br>*Only common tests should be described solely by name; describe more complex techniques in the Methods section.*
☒	☐	A description of all covariates tested
☒	☐	A description of any assumptions or corrections, such as tests of normality and adjustment for multiple comparisons
☐	☒	A full description of the statistical parameters including central tendency (e.g. means) or other basic estimates (e.g. regression coefficient) AND variation (e.g. standard deviation) or associated estimates of uncertainty (e.g. confidence intervals)
☐	☒	For null hypothesis testing, the test statistic (e.g. *F*, *t*, *r*) with confidence intervals, effect sizes, degrees of freedom and *P* value noted<br>*Give P values as exact values whenever suitable.*
☒	☐	For Bayesian analysis, information on the choice of priors and Markov chain Monte Carlo settings
☒	☐	For hierarchical and complex designs, identification of the appropriate level for tests and full reporting of outcomes
☐	☐	Estimates of effect sizes (e.g. Cohen's *d*, Pearson's *r*), indicating how they were calculated

Our web collection on statistics for biologists contains articles on many of the points above.

Software and code

Policy information about availability of computer code

Data collection	Animals were continuously recorded for off-line second-by-second scoring of freezing, measured manually by an observer blind of experimental groups
Data analysis	Data were analysed using the StatView software.

For manuscripts utilizing custom algorithms or software that are central to the research but not yet described in published literature, software must be made available to editors/reviewers. We strongly encourage code deposition in a community repository (e.g. GitHub). See the Nature Research guidelines for submitting code & software for further information.

Data

Policy information about availability of data

All manuscripts must include a data availability statement. This statement should provide the following information, where applicable:

- Accession codes, unique identifiers, or web links for publicly available datasets
- A list of figures that have associated raw data
- A description of any restrictions on data availability

All data is available in the main text or the supplementary information. Source Data that support the findings of this study are available from the corresponding author upon reasonable request. Source data that support the findings of this study are available at this address: 10.6084/m9.figshare.12644849.

Field-specific reporting

Please select the one below that is the best fit for your research. If you are not sure, read the appropriate sections before making your selection.

Life sciences study design

All studies must disclose on these points even when the disclosure is negative.

Sample size	We did not use a mathematical method for sample size calculation. Over the 15 years of experience since we first developped this task, we found that 8 mice per group is enough to reach statistical significance. To face the variability of surgery, we increased the sample size to 10 mice per group.<br>The power of these experiments, with n=8 mice per group and an alpha of 0.05, is 85%.
Data exclusions	Infection or implantation of optic fibers outside of the area shown within the dCA1 on Figure S1 was pre-established as incorrect and concerned mice were excluded from the study. The table in the methods section details the precise number of mice excluded, which were all in experiment 1.
Replication	All experiments were successfully replicated at least once.
Randomization	Every mice used in a single experiment were purchased from our provider from the same age group, at the same time. From this cohort, mice were randomly assigned to one experimental group, in order to avoid any "cage effect" (i.e. any event out of our control, (e.g. agressive male), which could have happened to one cage only among the total cohort)
Blinding	During all memory tests used in the study, animals were continuously recorded for off-line second-by-second scoring of freezing by an observer blind of experimental groups.

Reporting for specific materials, systems and methods

We require information from authors about some types of materials, experimental systems and methods used in many studies. Here, indicate whether each material, system or method listed is relevant to your study. If you are not sure if a list item applies to your research, read the appropriate section before selecting a response.

Materials & experimental systems

n/a	Involved in the study	
☒	☐	Antibodies
☒	☐	Eukaryotic cell lines
☒	☐	Palaeontology
☐	☒	Animals and other organisms
☒	☐	Human research participants
☒	☐	Clinical data

Methods

n/a	Involved in the study	
☒	☐	ChIP-seq
☒	☐	Flow cytometry
☒	☐	MRI-based neuroimaging

Animals and other organisms

Policy information about studies involving animals; ARRIVE guidelines recommended for reporting animal research

Laboratory animals	For all the experiments, 3-month-old naive male mice (C57Bl/6j, Charles River) were used.
Wild animals	The study did not involve wild animals.
Field-collected samples	No field-collected samples were used in the study.
Ethics oversight	All experimental procedures were conducted in accordance with the European Directive for the care and use of laboratory animals (2010-63-EU) and the animals care guidelines issued by the animal experimental committee of Bordeaux University (CCEA50, agreement number A33-063-099; authorization N°01377).

Note that full information on the approval of the study protocol must also be provided in the manuscript.
